# Split westerlies over Europe in the early Little Ice Age

**DOI:** 10.1038/s41467-022-32654-w

**Published:** 2022-08-20

**Authors:** Hsun-Ming Hu, Chuan-Chou Shen, John C. H. Chiang, Valerie Trouet, Véronique Michel, Hsien-Chen Tsai, Patricia Valensi, Christoph Spötl, Elisabetta Starnini, Marta Zunino, Wei-Yi Chien, Wen-Hui Sung, Yu-Tang Chien, Ping Chang, Robert Korty

**Affiliations:** 1 High-Precision Mass Spectrometry and Environment Change Laboratory (HISPEC), Department of Geosciences, National Taiwan University, Taipei, 10617 Taiwan ROC; 2 Research Center for Future Earth, National Taiwan University, Taipei, 10617 Taiwan ROC; 3 Department of Geography, University of California, Berkeley, CA 94720 USA; 4 Research Institute for Environmental Changes, Academia Sinica, Taipei, 11529 Taiwan ROC; 5 Laboratory of Tree-Ring Research, University of Arizona, Tucson, AZ 85721 USA; 6 Université Côte d’Azur, CNRS, CEPAM, Nice, 06300 France; 7 Université Côte d’Azur, CNRS, OCA, IRD, Géoazur, 06560 Valbonne, France; 8 HNHP, UMR 7194: CNRS-MNHN-UPVD, Paris, 75013 France; 9 Fondation IPH, Laboratoire de Préhistoire Nice-Côte d’Azur, Nice, 06300 France; 10 Institute of Geology, University of Innsbruck, Innsbruck, 6020 Austria; 11 Department of Civilizations and Forms of Knowledge, University of Pisa, Pisa, 56126 Italy; 12 Archaeological Superintendency of Liguria, Genova, 16126 Italy; 13 Toirano Cave, Piazzale D. Maineri 1, Toirano (SV), 17055 Italy; 14 National Science and Technology Center for Disaster Reduction, New Taipei City, 23143 Taiwan ROC; 15 Texas A&M University, College Station, TX 77843 USA

**Keywords:** Palaeoclimate, Water resources

## Abstract

The Little Ice Age (LIA; ca. 1450–1850 C.E.) is the best documented cold period of the past millennium, characterized by high-frequency volcanism, low solar activity, and high variability of Arctic sea-ice cover. Past studies of LIA Atlantic circulation changes have referenced the North Atlantic Oscillation (NAO), but recent studies have noted that LIA climate patterns appear to possess complexity not captured by an NAO analogue. Here, we present a new precipitation-sensitive stalagmite record from northern Italy that covers the past 800 years. We show that in the early LIA (1470–1610 C.E.), increased atmospheric ridging over northern Europe split the climatological westerlies away from central and northern Europe, possibly caused by concurrent Artic sea-ice reduction. With ongoing ice melting in the northern high latitudes and decreasing solar irradiance in the coming years, the early LIA may potentially serve as an analogue for European hydroclimatic conditions in the coming decades.

## Abstract (web summary)

A new stalagmite record from northern Italy and other published data from Europe and northern Africa reveals a split in the climatological westerlies during the early LIA, possibly attributed to sea ice melting.

## Introduction

The westerlies over the North Atlantic sector are the primary source of moisture transport to Europe, especially in winter half-year (October-March). A well-known influence on their path and strength is the pressure difference between the Icelandic Low and the Azores High; variations in this pressure difference give rise to the North Atlantic Oscillation (NAO; Supplementary Fig. 1A)^1^ and affect the precipitation patterns in Europe (Supplementary Fig. 1B). Another feature that gives rise to variation in the westerlies are atmospheric blocking events, i.e., persistent and stationary high-pressure systems that block the regular westerly flow for several days to weeks^2^. Atmospheric blocking over the North Atlantic sector and in particular Scandinavia plays an important role in extreme winter weather over Europe by modulating the trajectory of the westerlies and associated storm tracks (Supplementary Text 1)^3–6^. Their presence prevents the transport of warm and moist air masses, leading to cold spells in northern and central Europe such as the winter of 2010 C.E^7–9^. To better understand the variability of the westerlies and the occurrence of atmospheric blockings on different timescales, natural archives that extend back beyond the instrumental era are needed.

Several proxy records have recorded variability in the North Atlantic westerlies over the past millennia^10,11^ and especially during the Little Ice Age (LIA; ca. 1450–1850 C.E.)^12^. This was the coldest episode of the past millennium and featured low solar irradiance^13^, high variability in sea ice extent^14,15^, and frequent volcanic eruptions^16^. An early reconstruction of the NAO over the last millennium suggested a persistent positive NAO during the Medieval Climate Anomaly prior to a shift to negative NAO conditions during the LIA^10^, but this conclusion has since been questioned by other NAO reconstructions of the LIA^17,18^. A more recent multiproxy reconstruction suggests instead that the NAO was neutral to weak positive during the LIA^11^. There have been attempts to reconcile differing proxy reconstructions within the NAO framework^17–19^, but another possibility is that diverse patterns of large-scale atmospheric circulation may instead be at play.

Here, we present a new autumn-winter precipitation-sensitive stalagmite-based record from northern Italy that spans the past 800 years. Our record documents enhanced atmospheric ridging (anticyclone) over northern Europe accompanied by a split in the climatological westerlies, with the main branch extending towards the Mediterranean and a weaker branch northward towards Greenland. This circulation is characteristic of a pronounced positive phase of the Scandinavian teleconnection pattern, a mode of wintertime large-scale atmospheric circulation variability over the North Atlantic and Europe that is dynamically distinct from the NAO^20–25^.

## Results

Bàsura cave (44.13 ˚N, 8.2 ˚E, 200 m above sea level [a.s.l.]), featuring a Mediterranean climate with dry summers and humid winters, is located in Toirano, northern Italy (Supplementary Fig. 2). Instrumental data from Genoa meteorological station (44.41 ˚N, 8.93 ˚E, 55 m a.s.l., 70 km northeast of Bàsura cave; 1950–2008 C.E.) show that more than 70% of the annual precipitation of 1269 ± 331 mm (1-sigma) falls during the rainy season from September to February (Sep–Feb). The interior of the 1 km-long cave is characterized by 97–100% relative humidity and a stable annual temperature of 15.6 ˚C. Stalagmite BA18-4 (Supplementary Fig. 3A) was collected in a narrow chamber 350 m from the main entrance in 2018 C.E for Mg/Ca, Sr/Ca and Ba/Ca analysis and U-Th dating (Methods). X-ray diffraction analysis shows that this stalagmite is composed of calcite.

Over the past 800 years, Bàsura Mg/Ca, Sr/Ca, and Ba/Ca vary between 20–30 mmol/mol, 0.035–0.055 mmol/mol, and 4.0–8.0 μmol/mol, respectively (Supplementary Fig. 4). The trace element/calcium (TE/Ca) ratio in stalagmites is a proxy of hydroclimate above the cave modulated by prior carbonate precipitation (PCP)^26–31^. PCP, i.e., precipitation of carbonate in the karst aquifer before the dripwater reaches the stalagmite, is enhanced during dry climate conditions due to reduced recharge, long residence time, and low CO_2_ concentration in the cave air. Covariation of Mg/Ca, Sr/Ca, and Ba/Ca ratios in dripwater and in the stalagmite suggests a strong PCP effect when the partition coefficients (D_Me_ = (TE/Ca)_calcite_/(TE/Ca)_dripwater_) of these elements are less than one^29–31^. In the Bàsura stalagmite BA18-4, Ba/Ca and Sr/Ca ratios are strongly positively correlated (*r*^2^ = 0.90, *n* = 230, *p* < 0.01, Supplementary Fig. 5A), indicating a strong PCP effect^26–31^. A lower correlation coefficient (*r*^2^ = 0.59, *n* = 232, *p* < 0.01) for Mg/Ca versus Sr/Ca (Supplementary Fig. 5b) suggests additional controls on Mg/Ca (Supplementary Text 2).

This inconsistency between Mg/Ca and Sr/Ca can be potentially attributed to a temperature effect on Mg/Ca^26–31^. For example, Northern Hemisphere temperature records (Supplementary Fig. 4a)^32^ and Bàsura Mg/Ca (Supplementary Fig. 4b) show a clear decreasing multidecadal trend from 1500 to 1600 C.E. and an increasing trend from 1850 C.E. onwards, suggesting a temperature effect on stalagmite Mg/Ca, with high Mg/Ca corresponding to high temperature. Stalagmite Mg/Ca variations were also proposed to be affected by the source effect in the Mediterranean region^33^. Strong westerly winds could lead to the deposition of Mg-enriched particles (derived from dolomite-dominated coastal regions) in the catchment of the cave, which results in high Mg/Ca ratio in the speleothem^33^. Compared to Mg/Ca, Sr/Ca and Ba/Ca are less influenced by temperature^26–29^ and thus are more suitable for reconstructing paleohydrology. Bàsura Sr/Ca is significantly negatively correlated (*r* = – 0.63, *n* = 36, *p* < 0.05) with instrumental Sep–Feb (rainy seasons) precipitation records from the weather stations of Genoa (G; 44.41 ˚N, 8.93 ˚E, 55 m a.s.l.), Milan (M; 45.47 ˚N, 9.19 ˚E, 150 m a.s.l.), and Nice (N; 43.65 ˚N, 7.21 ˚E, 2 m a.s.l.) for 1855-1965 C.E. within dating uncertainties (Supplementary Fig. 6; Supplementary Text 2). This linkage is supported by in situ dripwater monitoring results, which show deceasing of dripwater Sr/Ca and Ba/Ca corresponding to intervals with high rainfall (Supplementary Text 2). We therefore use the Bàsura stalagmite Sr/Ca record to represent Toirano autumn-winter (Sep–Feb) precipitation history.

### Southern European precipitation pattern

**Fig. 1:**
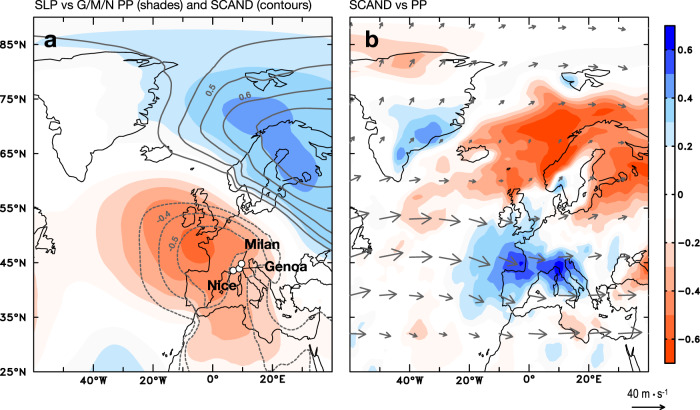
Climate and Atlantic sea-level pressure (SLP) variability. **a** Correlation between SLP and (i) average precipitation at Genoa, Milan and Nice stations (G/M/N PP) (shades); (ii) Climate Prediction Center (CPC) Scandinavia index (SCAND) (contours) during September–February in 1950–2008 C.E. **b** Vectors: climatological winds at 200-mb level plus regression of 200-mb winds on SCAND index multiplied by two standard deviations of SCAND index during September-February in 1950–2008 C.E., indicating a positive SCAND condition. Shades: correlation between SCAND and ground precipitation during September-February in 1950–2008 C.E. The shades and contours indicate the correlation coefficient(s) above 90% confident level. Climate data are from 20^th^ century reanalysis v3.

Toirano autumn-winter precipitation is strongly related to variability in autumn-winter North Atlantic sea-level pressure. Instrumental autumn-winter precipitation data averaged for the G/M/N stations (1950–2008 C.E.) show a strong positive correlation with sea-level pressure (SLP) anomalies, with a ridge over Scandinavia and a trough over western Europe (Fig. 1a, shades). The correlation pattern closely resembles the SLP pattern associated with the Scandinavian teleconnection (SCAND; Fig. 1a, contours)^6^, and is distinguished from NAO-correlation pattern (Supplementary Fig. 1a and c). Indeed, G/M/N Sep–Feb precipitation is strongly positively correlated with an index of the Scandinavian pattern (SCAND index; https://www.cpc.ncep.noaa.gov/data/teledoc/scand.shtml) averaged over Sep–Feb (*r* = 0.63, *n* = 70, *p* < 0.05, 1950–2020 C.E.), suggesting that SCAND exerts a strong control on autumn-winter precipitation in Toirano. Positive winter SCAND phases are associated with synoptic high-pressure anomalies over eastern Scandinavia and low-pressure anomalies over western Europe. Wind field analysis (Fig. 1b; vectors) shows that a positive SCAND index is associated with a split configuration of the climatological westerlies, with the northern branch extending towards Greenland and the southern branch flowing into the Mediterranean. The altered moisture transport by the changing westerlies affects the hydroclimate in Greenland and the Mediterranean region (Fig. 1b, shades), with increasing precipitation in the northern Mediterranean and SE Greenland.

In the observed wintertime synoptic circulation over the Euro-Atlantic sector, Scandinavia is a preferred location for atmospheric blocking and the associated blocking high structure produce a bifurcation in the storm track leading to increased precipitation over the southernmost Europe, northernmost of Norway, and SE Greenland (Supplementary Fig. 1d)^20^. An increase in the frequency of atmospheric blocking events over Scandinavia would thus lead on climate timescales to atmospheric ridging, split climatological westerlies and associated rainfall changes as expressed by the positive SCAND pattern. Given the demonstrated modern-day relationship between Toirano rainfall and the SCAND pattern (Fig. 1b), we thus argue that Bàsura stalagmite Sr/Ca and Ba/Ca record the frequency of wintertime atmospheric blocking events over Scandinavia, with more rainfall indicating an increased occurrence of blocking and split westerlies over the Europe.

### Little Ice Age

**Fig. 2:**
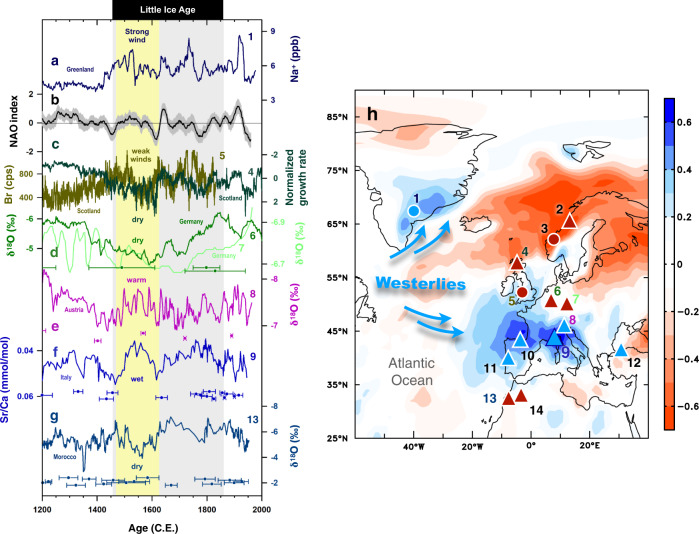
Climate records from Europe and northern Africa for the last 800 years. **a** Na^+^ concentration in ice core GISP2 as an indicator of wind strength^34^. **b** Reconstructed North Atlantic Oscillation (NAO) index^11^. **c** Green: Stalagmite growth rate from Roaring cave (Scotland) as a proxy of precipitation amount. Low growth rate indicates positive NAO phase^35^. Mustard: Bromine concentration from aeolian sediments as a proxy of wind strength. High values indicate strong winds^36^. **d** Bunker (dark green) and Bleßberg (light green) δ^18^O record from Germany^37–39^. Low values reflect a warm and wet climate. **e** Spannagel δ^18^O record from Austria^42^. Low values denote a warm climate. **f** Bàsura Sr/Ca record from northern Italy. **g** Ifoulki δ^18^O record from Morocco^46^. Grey vertical band marks the Little Ice Age. The yellow vertical bar highlights the period 1470-1610 C.E. Colored-coded dots and bars are U-Th ages with 2-sigma uncertainties. **h** Map showing the climate configuration and location of the cited records. The red-blue shades show the correlation coefficient(s) between Scandinavia index and ground precipitation during September-February in 1950–2008 C.E. Climate data are from 20^th^ century reanalysis v3. Triangles and circles mark westerly-affected sites with a wet/warm (blue) or dry/cold (red) climate. The Bàsura cave is highlighted by a dark blue edge. 1: GISP2^34^. 2: Korallgrottan cave^40^. 3: Neflon^41^. 4: Roaring cave^35^. 5: Outer Hebrides^36^. 6: Bunker cave^37,38^. 7: Bleßberg cave^39^ 8: Spannagel cave^42^. 9. Bàsura cave. 10: Kaite cave^43^. 11: Buraca Gloriosa cave^43^. 12: Sofular cave^45^. 13: Ifoulki cave^34^. 14: Chaara cave^47^.

During the LIA, the climate in the North Atlantic/European realm was mostly cold^12^, but proxy records show considerable variability on multi-decadal to centennial time-scales within this period. On centennial time-scales, the Bàsura record reveals a distinct wet interval in northern Italy during the early LIA (1470–1610 C.E.), suggesting possible strong and persistent positive SCAND-like conditions. Within this period, however, the Bàsura record (Fig. 2f) shows strong multi-decadal variability: precipitation increased at the beginning of the LIA, peaked in ~1550 C.E., and then decreased until 1620 C.E. Precipitation reached a second peak in the late 1700 s, followed by a decreasing trend until the end of the LIA.

The early LIA wet interval in northern Italy corresponds to a period of strong westerlies over the North Atlantic (1470–1610 C.E.), as suggested by a sodium ion record in a Greenland ice core (Fig. 2a)^34^, concurrent with a neutral-positive NAO mode as indicated by a composite NAO reconstruction (Fig. 2a)^11^. The British Isles, however, experienced a relatively dry and less windy climate during this interval, as recorded in a stalagmite-based reconstruction of Roaring cave (Fig. 2c, green)^35^ and the aeolian sediment Bromine record from Scotland (Fig. 2c; mustard)^36^. Dry and cold conditions are also inferred for Germany, based on stalagmite δ^18^O data from Bunker cave (Fig. 2d, dark green)^37,38^ and Bleßberg cave (Fig. 2d, light green)^39^, and for high-latitude Sweden (Supplementary Fig. 7a)^40^, based on stalagmite δ^18^O data and lacustrine records^41^. The precipitation and wind minima in these regions thus do not reflect a neutral-positive NAO mode^35^. Our Sr/Ca record from Bàsura cave (Fig. 2f), on the other hand, suggests that southern Europe experienced warm and humid conditions, as also documented by a stalagmite δ^18^O record from Spannagel cave, Austria (Fig. 2e)^42^. Similarly, warm and wet conditions are registered by stalagmite records from Portugal (Supplementary Fig. 7b)^43^, Spain (Supplementary Fig. 7c)^44^, and Turkey (Supplementary Fig. 7d)^45^, whereas stalagmite records from Morocco record a dry interval (Fig. 2g and Supplementary Fig. 7f)^46,47^.

### Atmospheric ridging over Scandinavia during 1470–1610 C.E.

The dry/cold climate in northern Europe during 1470–1610 C.E. can be reconciled with a neutral-positive NAO phase during this period as suggested by Ortega et al. (2015)^11^ through increased atmospheric ridging and hence increased frequency of atmospheric blocking events over Scandinavia. Comas-Bru and McDermott (2013)^22^ have argued that the additional influence of the SCAND pattern on top of an NAO can explain the nonstationarity in the relationship between European winter climate and the NAO. The combination of the Greenland record and our Bàsura record suggests that such ridging split the westerlies during the early LIA, with a northern branch directed towards the Arctic, consistent with the windy/warm/humid climate over southeastern Greenland, and a southern branch directed towards the Mediterranean, consistent with increased rainfall at Bàsura cave (Fig. 2h). This climatic setting is similar to the anomalously wet conditions in southeastern Greenland and the northwestern Mediterranean during positive SCAND phases (Fig. 1b and Supplementary Fig. 1d)^4,48^. The positive SCAND phase does not explain the early LIA drying over North Africa^46,47^, but this could be attributed to an enhanced Azores High during a neutral-positive NAO phase (Fig. 2a)^11^, which prevented moisture transport into Morocco (Fig. 2b).

### Connections to reduced Arctic sea ice and solar forcing

**Fig. 3:**
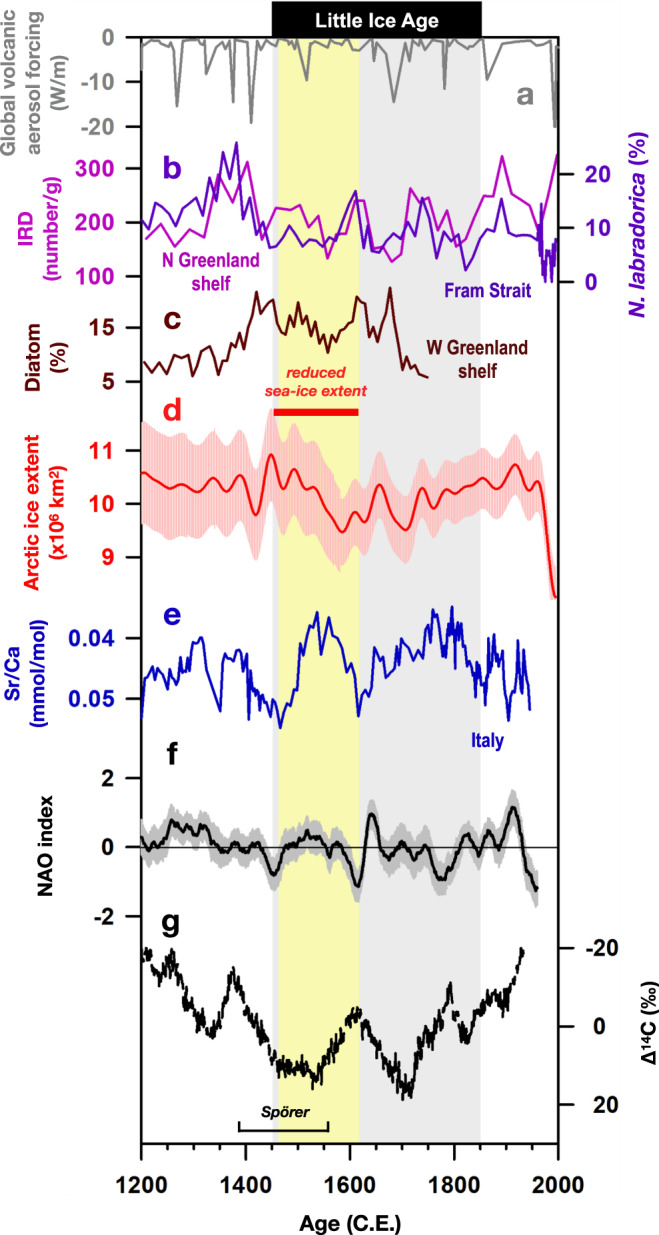
Comparison of volcanic forcing, solar activity, sea-ice variability, and Bàsura record. **a** Global volcanic aerosol forcing^16^. **b** Violet: concentration of benthic foraminifera from the North Greenland shelf (PS2641-4; Supplementary Fig. 2) as an indicator of sea-ice cover^50^. Pink: ice-rafted debris (IRD) (MSM5/5; Supplementary Fig. 2) from the Fram Strait^49^. High values of these two records denote large sea-ice cover. **c** Five-point averaged diatom concentration (*Thalassiosira nordenskioeldii*) from the west Greenland shelf (GA306-4; Supplementary Fig. 2)^51^. High value denotes large sea-ice cover. **d** Red: 40-year smoothed reconstructed late summer Arctic sea-ice extent^56^. **e** Bàsura Sr/Ca record. **f** Reconstructed NAO index^11^. **g** Total solar irradiance^12^. The intervals of the Spörer Minimum (1388–1558 C.E.)^12^ and decreased sea-ice event (1450–1620 C.E.)^56^ are marked. The grey vertical bar denotes the Little Ice Age. The yellow vertical bar highlights the period 1470–1610 C.E.

Ice-rafted debris (IRD) records from the Fram Strait (Fig. 3b)^49^, foraminiferal-inferred sea-ice records on the North Greenland shelf (Fig. 3b)^50^, and diatom-based sea-ice reconstructions from the west Greenland shelf (Fig. 3c)^51^ all show extensive sea-ice cover in the North Atlantic during the middle to late 1300 s. This extensive sea-ice cover in the North Atlantic was presumably induced by intense volcanism^16^ (Fig. 3a) and low solar irradiance^12^ (Fig. 3g) in the late 1200 s to early 1300 s (refs. 14,52) and in turn it triggered a change in ocean circulation and cooling in Europe starting around 1400 C.E. (ref. 15,53). From the 1400 s C.E. onwards, sea ice extent decreased significantly (Fig. 3b, c), possibly related to sea-ice conditions^54,55^. Considering the dating uncertainties of major Bàsura Sr/Ca decreases between 1466 ± 20 and 1559 ± 40 C.E., the period with Scandinavian ridging we inferred falls in the interval with Artic sea-ice maximum at 1450 C.E. to minimum at 1586 C.E., as this interval was identified as a “decreased sea ice extent” event^56^. Such Scandinavian ridging can be triggered by sea ice loss in the Barent-Kara seas^57,58^. While direct sea-ice proxy records from Barent-Kara seas are not available, coupled model simulations constrained by the assimilation of available global proxy data indicate a reduction of Barent-Kara sea ice during 1470–1520 C.E^59^. Reduced sea ice results in increased heat fluxes into the atmosphere that, in turn, excite a stationary Rossby wave propagating towards the southeast and can increase atmospheric ridging over northern Europe^60–62^. This ocean-atmosphere feedback could thus provide a mechanism for the link between reduced sea-ice extent and the Scandinavian ridging during 1470–1610 C.E. suggested by our results (Fig. 2g).

The atmospheric ridging in the early LIA could have been further amplified by low solar irradiation (Fig. 3g). Model simulations^63,64^ and proxy records^65^ suggest that solar irradiation changes can have a significant effect on ozone chemistry in the stratosphere that disturbs the polar vortex and thus influences the tropospheric jet stream and atmospheric circulation^63–67^. Our Bàsura stalagmite Sr/Ca data, decreasing from 0.055 mmol/mol at 1460 C.E. to 0.035 mmol/mol at 1550 C.E., suggest that Scandinavian ridging progressively strengthened during this interval (Fig. 3e). This 90-yr interval falls during the Spörer Minimum (1388–1558 C.E., Fig. 3g)^12^, supporting this linkage between atmospheric changes and solar variability^68^.

### Complex climate patterns in the late LIA

During the second-half of the LIA, the Bàsura record shows a wetting trend from 1610 to 1750 C.E., similar to records from Bunker cave (Germany; Fig. 2d)^37,38^, Sofular cave (Turkey; Supplementary Fig. 7d)^45^, and Chaara cave (Morocco; Supplementary Fig. 7f)^46^. However, records from Roaring cave (Scottland; Fig. 2c)^35^, Ifoulki cave (Morocco; Fig. 2g)^34^ and Kaite cave (Spain; Supplementary Fig. 7c)^43^ show an opposite trend, whereas there is no clear trend in the records from Spannagel cave (Austria; Fig. 2e)^42^. The diverse climatic conditions during the second half of the LIA suggest that this period could be more complex than the first half. The NAO reconstruction (Fig. 2b)^11^ also shows a neutral phase for the late LIA with trivial fluctuations, suggesting that the NAO did not play an important role in orchestrating European climate during this period. The NAO, Scandinavian patterns, and/or other leading climate modes (such as the East Atlantic pattern)^6^ could contribute equally (or weakly) to late LIA European climate, which leads to complex regional precipitation patterns over Europe.

The decreased sea ice extent from 1450–1620 C.E. arguably shows a long duration over the past 1400 years and is more pronounced than that during the Medieval Climate Anomaly (ca. 800–1300 C.E.)^56^. Our results present proxy-based evidence of enhanced atmospheric ridging over northern Europe during this multi-decadal interval at the early LIA, possibly in response to the sea ice reduction and solar minimum. Our results thus potentially provide an analogue for the coming decades, when the sun could enter a grand minimum^69^ and the Arctic is projected to be ice free by 2030 C.E^70^.

## Methods

Chronology of BA18-4 was established using StalAge^71^ based on 15 ^230^Th dates, measured on a Thermo-Finnigan Neptune multi-collector inductively coupled plasma mass spectrometer^72^ at National Taiwan University, with a two-sigma dating uncertainty of ± 4 to 37 years (Supplementary Fig. 3B). A total of 214 subsamples were drilled for Mg, Sr, and Ba and analyzed using external matrix-matched in-house standards for every 4-5 samples on an inductively coupled plasma sector-field mass spectrometer (ICP-SF-MS, Finnigan Element II)^73^ (Supplementary Data 1) at National Taiwan University, with a two-sigma reproducibility of ± 0.5%.

## Supplementary information

Supplementary Information

Description of Additional Supplementary Files

Supplementary Data 1

## Data Availability

The trace element data generated in this study are provided in the Source Data file.
