# Author Correction: Split westerlies over Europe in the early Little Ice Age

**DOI:** 10.1038/s41467-022-33567-4

**Published:** 2022-10-10

**Authors:** Hsun-Ming Hu, Chuan-Chou Shen, John C. H. Chiang, Valerie Trouet, Véronique Michel, Hsien-Chen Tsai, Patricia Valensi, Christoph Spötl, Elisabetta Starnini, Marta Zunino, Wei-Yi Chien, Wen-Hui Sung, Yu-Tang Chien, Ping Chang, Robert Korty

**Affiliations:** 1 High-Precision Mass Spectrometry and Environment Change Laboratory (HISPEC), Department of Geosciences, National Taiwan University, Taipei, 10617 Taiwan ROC; 2 Research Center for Future Earth, National Taiwan University, Taipei, 10617 Taiwan ROC; 3 Department of Geography, University of California, Berkeley, CA 94720 USA; 4 Research Institute for Environmental Changes, Academia Sinica, Taipei, 11529 Taiwan ROC; 5 Laboratory of Tree-Ring Research, University of Arizona, Tucson, AZ 85721 USA; 6 Université Côte d’Azur, CNRS, CEPAM, Nice, 06300 France; 7 Université Côte d’Azur, CNRS, OCA, IRD, Géoazur, 06560 Valbonne, France; 8 HNHP, UMR 7194: CNRS-MNHN-UPVD, Paris, 75013 France; 9 Fondation IPH, Laboratoire de Préhistoire Nice-Côte d’Azur, Nice, 06300 France; 10 Institute of Geology, University of Innsbruck, Innsbruck, 6020 Austria; 11 Department of Civilizations and Forms of Knowledge, University of Pisa, Pisa, 56126 Italy; 12 Archaeological Superintendency of Liguria, Genova, 16126 Italy; 13 Toirano Cave, Piazzale D. Maineri 1, Toirano (SV), 17055 Italy; 14 National Science and Technology Center for Disaster Reduction, New Taipei City, 23143 Taiwan ROC; 15 Texas A&M University, College Station, TX 77843 USA

**Keywords:** Palaeoclimate, Water resources

**Correction to**: *Nature Communications* 10.1038/s41467-022-32654-w, published online 20 August 2022

The original version of this Article contained an error in Fig. 3g, in which the age on the X axis for the Atlantic Multidecadal Variability index record was incorrect. This has been corrected in both the PDF and HTML versions of the Article.

