# Author Correction: Split westerlies over Europe in the early Little Ice Age

**DOI:** 10.1038/s41467-023-37371-6

**Published:** 2023-03-22

**Authors:** Hsun-Ming Hu, Chuan-Chou Shen, John C. H. Chiang, Valerie Trouet, Véronique Michel, Hsien-Chen Tsai, Patricia Valensi, Christoph Spötl, Elisabetta Starnini, Marta Zunino, Wei-Yi Chien, Wen-Hui Sung, Yu-Tang Chien, Ping Chang, Robert Korty

**Affiliations:** 1 High-Precision Mass Spectrometry and Environment Change Laboratory (HISPEC), Department of Geosciences, National Taiwan University, Taipei, 10617 Taiwan ROC; 2 Research Center for Future Earth, National Taiwan University, Taipei, 10617 Taiwan ROC; 3 Department of Geography, University of California, Berkeley, CA 94720 USA; 4 Research Institute for Environmental Changes, Academia Sinica, Taipei, 11529 Taiwan ROC; 5 Laboratory of Tree-Ring Research, University of Arizona, Tucson, AZ 85721 USA; 6 Université Côte d’Azur, CNRS, CEPAM, Nice, 06300 France; 7 Université Côte d’Azur, CNRS, OCA, IRD, Géoazur, 06560 Valbonne, France; 8 HNHP, UMR 7194: CNRS-MNHN-UPVD, Paris, 75013 France; 9 Fondation IPH, Laboratoire de Préhistoire Nice-Côte d’Azur, Nice, 06300 France; 10 Institute of Geology, University of Innsbruck, Innsbruck, 6020 Austria; 11 Department of Civilizations and Forms of Knowledge, University of Pisa, Pisa, 56126 Italy; 12 Archaeological Superintendency of Liguria, Genova, 16126 Italy; 13 Toirano Cave, Piazzale D. Maineri 1, Toirano (SV), 17055 Italy; 14 National Science and Technology Center for Disaster Reduction, New Taipei City, 23143 Taiwan ROC; 15 Texas A&M University, College Station, TX 77843 USA

**Keywords:** Palaeoclimate, Water resources

Correction to: *Nature Communications* 10.1038/s41467-022-32654-w, published online 20 August 2022

The original version of this Article contained an error in Fig. 3, in which the uncertainty of the Atlantic Multidecadal Variability index was included as an orange trend line labelled with the letter ‘g’. This index however was found to be not linked to the findings of this study and has been removed. This has been corrected in both the PDF and HTML versions of the Article.

The original version of this Article contained an error in the caption of Figure 3, which incorrectly read ‘g Orange: Atlantic Multidecadal Variability index^55^. Black: total solar irradiance^12^.’ The correct version replaces this sentence with ‘g Total solar irradiance^12^.’. This has been corrected in both the PDF and HTML versions of the Article.

The original version of this Article contained an error in the Results section, which incorrectly read ‘From the 1400 s C.E. onwards, sea ice extent decreased significantly (Fig. 3b, c) due to an intrusion of warm Atlantic Water into the North Atlantic^54^, as reflected by a positive phase of the Atlantic Multidecadal Variability (Fig. 3g)^55^.’ The correct version replaces this sentence with ‘From the 1400 s C.E. onwards, sea ice extent decreased significantly (Fig. 3b, c), possibly related to sea-ice conditions^54,55^.’. This has been corrected in both the PDF and HTML versions of the Article.

