# Supplementary material for: Split westerlies over Europe in the early Little Ice Age
Source: Nat Commun. 2022 Aug 20;13:4898. doi: 10.1038/s41467-022-32654-w (PMC9392774; doi:10.1038/s41467-022-32654-w)
Supplement: Supplementary file 1 — Supplementary Information [file 41467_2022_32654_MOESM1_ESM.pdf]

Supplementary Information for

Split westerlies over Europe in the early Little Ice Age

Hsun-Ming Hu, Chuan-Chou Shen, John C. H. Chiang, Trouet Valerie, Véronique Michel, Hsien-Chen Tsai, Patricia Valensi, Christoph Spötl, Elisabetta Starnini, Marta Zunino, Wei-Yi Chien, Wen-Hui Sung, Yu-Tang Chien, Ping Chang and Robert Korty

Correspondence to: Chuan-Chou Shen (river@ntu.edu.tw)

This PDF file includes:

Supplementary Texts 1 to 2

Supplementary Figures 1 to 8

Other Supplementary Materials for this manuscript include the following:

Supplementary Data 1

Supplementary Text

1. Atmospheric blockings

The term 'atmospheric blocking' describes a synoptic anticyclonic feature in the midlatitude atmosphere that persists on timescales of days to weeks, and acts to "block" the westward trajectory of midlatitude jet streams and associated storm tracks[2]. Atmospheric blockings occur in well-defined regions, such as the North Pacific off the west coast of North America or Atlantic, and are observed over oceans and land[7-9] in European realm. Blocking over the Euro-Atlantic sector further leads to a reduction in rainfall over most of Europe except for its northernmost and southernmost sectors where rainfall increases due to a bifurcation of the storm track around the blocking high[20]. In our study, the term 'atmospheric blocking' is used to indicate features on synoptic time-scales and the term 'atmospheric ridging' to indicate seasonal-to-centennial time scales.

2. Interpretation of Sr/Ca and Ba/Ca

Stalagmite trace element/Ca (TE/Ca) ratios vary under differing regional and cave conditions and hydroclimates[26-31]. In general, stalagmite Sr/Ca and Ba/Ca are considered as indicators of cave hydrology variations through the mechanism of prior carbonate precipitation (PCP)[26-31]. Dry conditions are expected to enhance CO_2 degassing and result in a long residence time of the infiltrated water in the epikarst, leading to high TE/Ca because of the preferential removal of Ca during PCP. However, the Sr/Ca water-carbonate distribution coefficients (D_{Sr}), $(Sr/Ca)_{carbonate}$ /$(Sr/Ca)_{water}$, are 0.1–0.2 for calcite[30] and 0.8–2.0 for aragonite[31]. These levels would lead to possible Sr/Ca changes in response to hydroclimate in both negative ($D_{Sr} < 1$) and positive ways ($D_{Sr} > 1$) if the phase of PCP is unknown. A second TE/Ca ratio, i.e., Ba/Ca in our case, is used to support the calcite Sr/Ca interpretation[31]. A concurrent enrichment/depletion of Sr/Ca and Ba/Ca suggests a common source change, since the distribution coefficient of Ba (D_{Ba}) is less than one either in calcite or aragonite. While the growth rate and temperature changes affect the Ba/Ca ratio, their effects are insignificant in Sr/Ca[30]. Accordingly, the general consistency of Sr/Ca and Ba/Ca is often used as an indicator of PCP[26-29] and hence can be used as the precipitation indicator.

Bàsura Sr/Ca record was tuned to September to February precipitation time series from the weather stations of Genoa, Milan, and Nice for 1855-1965 C.E. and yields a correlation coefficient of – 0.63 ($n = 36$, $p < 0.05$) (Supplementary Figure 6). The tuning was done within age uncertainties derived from StalAge[72] age model for BA18-4. Tuning certainly increases the likelihood of finding significant correlations because the general dating uncertainties during this period (1855-1965 C.E.) could be up to ± 20 years and only two U-Th dating controls were presented after 1900 C.E. Noted the significant correlation between the Sr/Ca and instrument data could be lowered down if future age model was improved. Despite of the limitation of the correlation analysis, in-situ monitoring of Sr/Ca and Ba/Ca ratios of dripwater can provide supportive evidence for our interpretations of BA18-4 Sr/Ca and Ba/Ca ratios. Lows in dripwater Sr/Ca and Ba/Ca match high rainfall events, recorded at the Cape-Mele meteorological station, [43.95 ˚N, 8.16 ˚E], 20 km south of Bàsura cave, on 2019/11, 2020/06, and 2020/12-2021/01 (Supplementary Figure 8). Even though this monthly data cannot be used as a direct analogue for the records on multi-annul or longer timescales, they demonstrate that the dripwater generally features low Sr/Ca and Ba/Ca ratios when precipitation increased. Over decadal to centennial timescales, the enhanced precipitation increased over a year (or a longer interval) leads to an increasing proportion of dripwater with low Sr/Ca and Ba/Ca, eventually registered in stalagmites. Low Sr/Ca and Ba/Ca in the Bàsura stalagmite record can therefore suggest a wet climate.

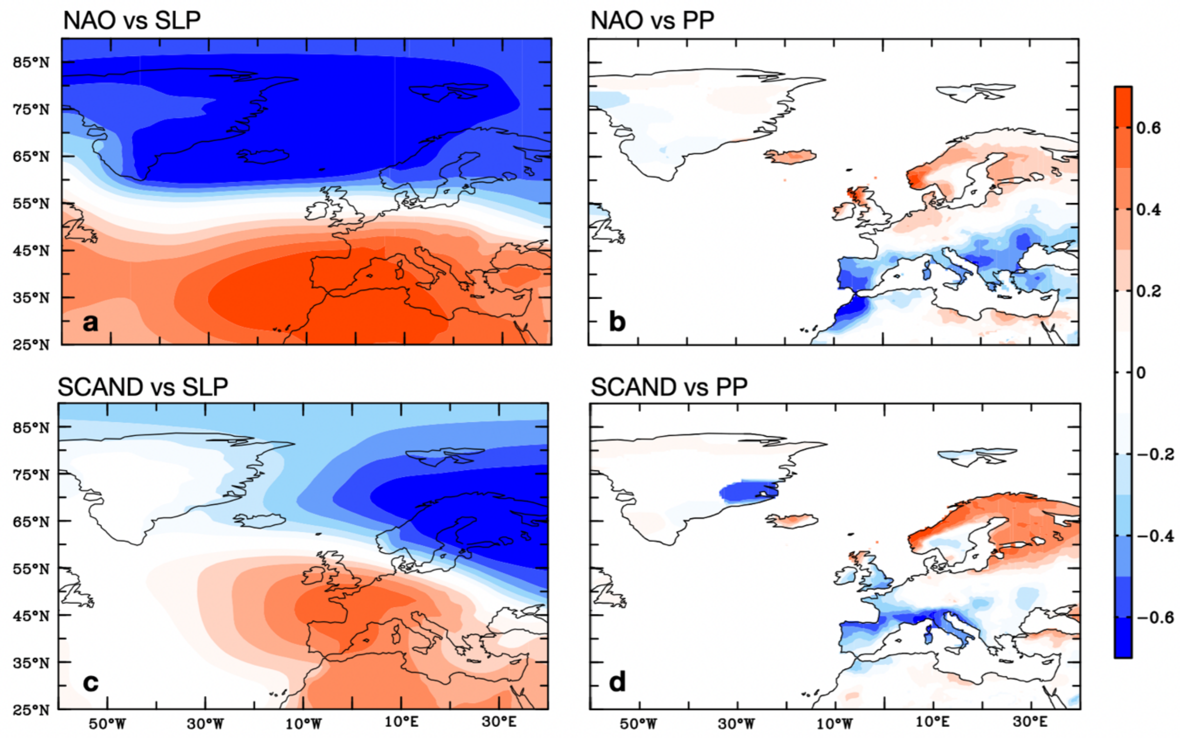

Supplementary Figure 1. **Correlation analysis.** Correlation analysis for **(a)** North Atlantic Oscillation (NAO) index and sea-level pressure (SLP), **(b)** NAO index and precipitation (PP), **(c)** Scandinavian (SCAND) index and SLP, and **(d)** SCAND index and PP. Shades: correlations between SCAND and ground precipitation during September-February during 1950–2008 C.E. The shades and contours indicate the correlation coefficient(s) above 90% confident level. Climate data are from 20 century reanalysis v3 for SLP and from CRU TS Version 4.04.

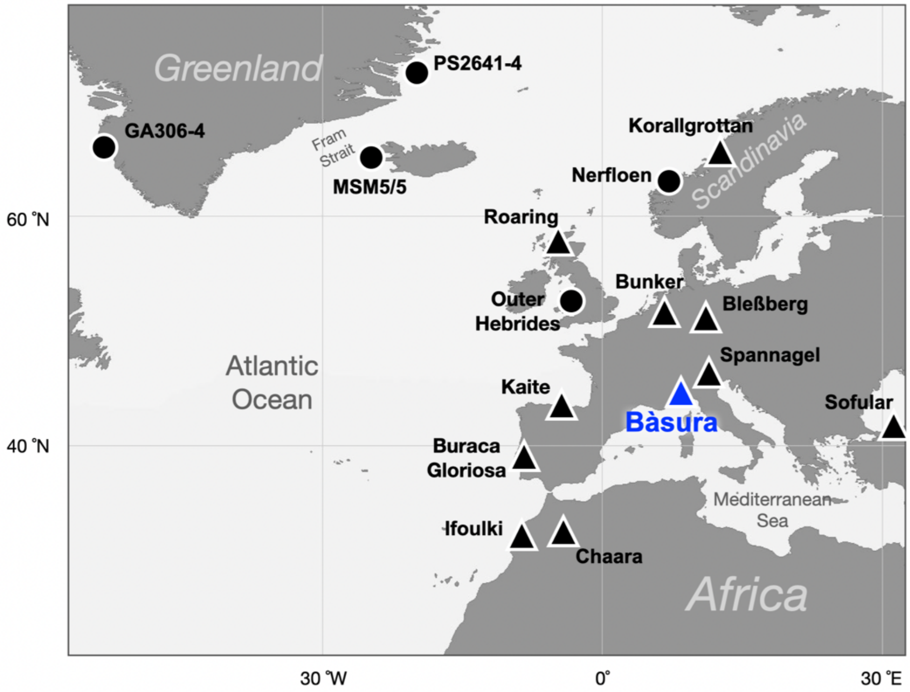

Supplementary Figure 2. Map of the study area. Triangles (caves) and circles (lake/marine cores) are the sites mentioned in the main text. The blue triangle highlights Bàsura cave. This map is generated from Ocean Data View.

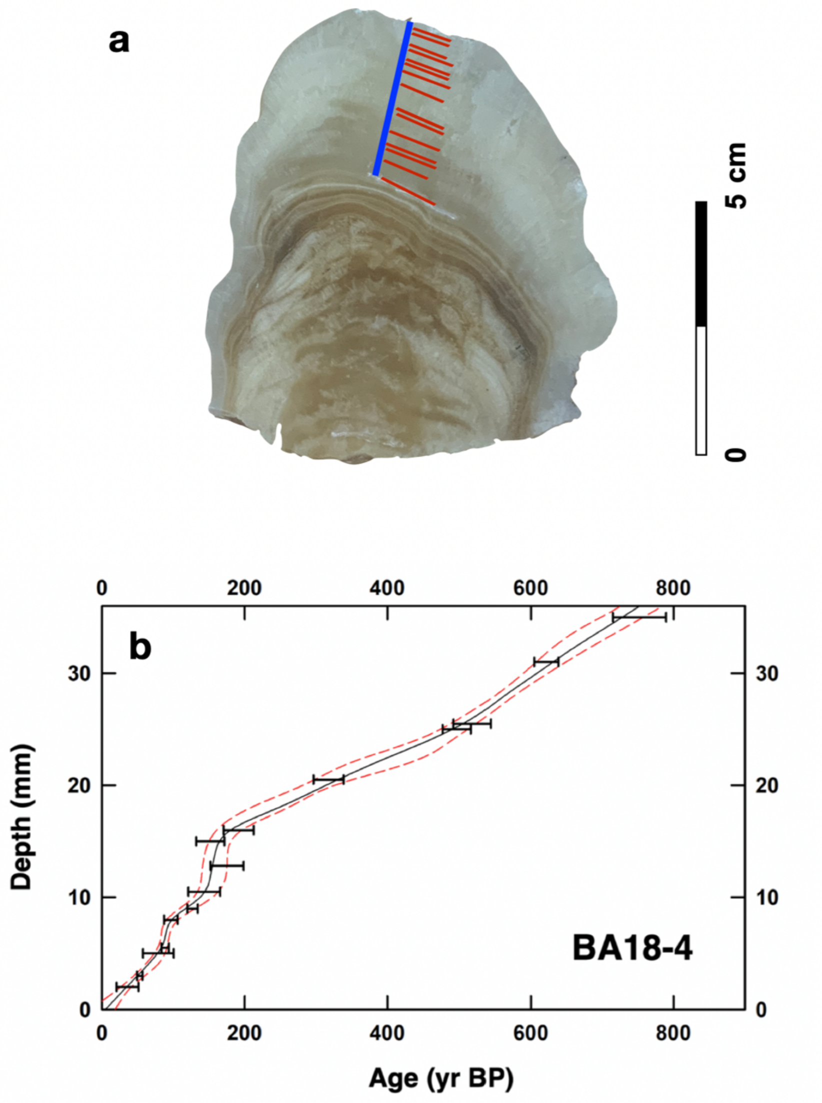

Supplementary Figure 3. Photography of BA18-4 and age model. (a) Polished slab of stalagmite BA18-4. The blue and red lines denote the positions of subsamples for trace element analysis and ^{230}Th dates, respectively. **(b)** Age model of BA18-4 built with ^{230}Th dates by StalAge method[72].

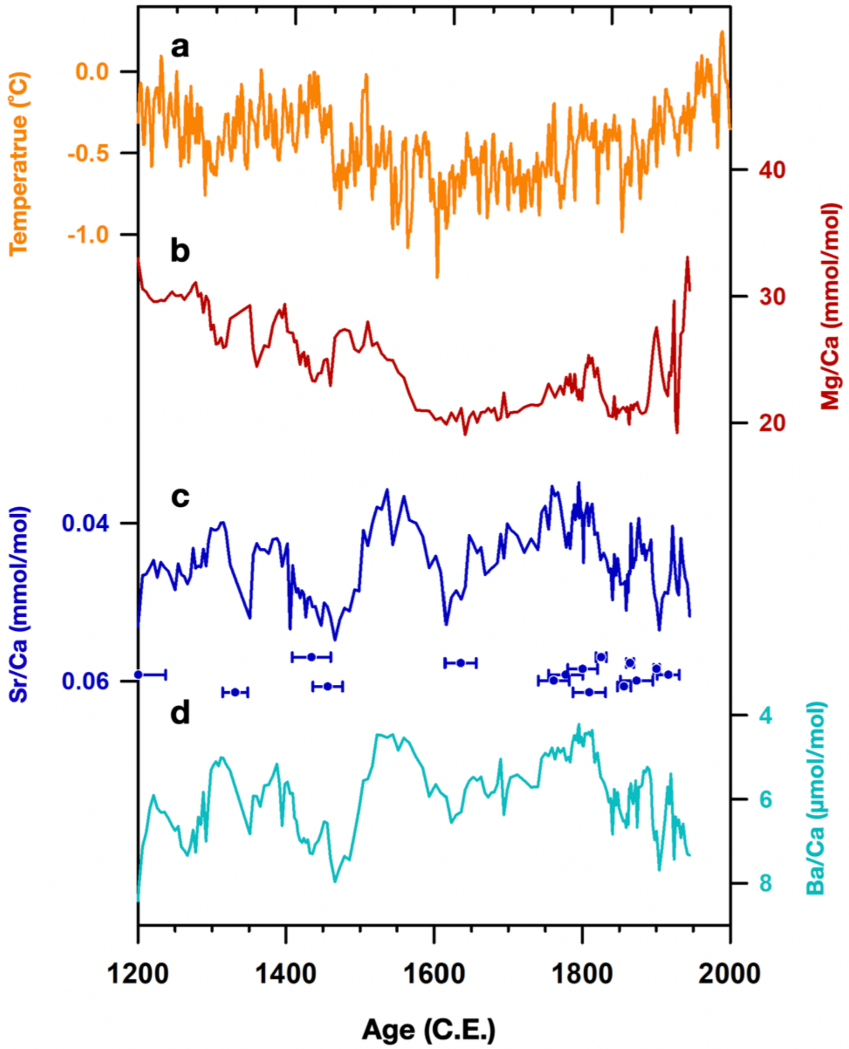

Supplementary Figure 4. Comparison of temperature and Bàsura trace element data. (a) Northern Hemisphere temperature records[32]. Bàsura 18-4 stalagmite **(b)** Mg/Ca, **(c)** Sr/Ca, and **(d)** Ba/Ca records. Error bars indicate ^{230}Th dates with 2-sigma uncertainties.

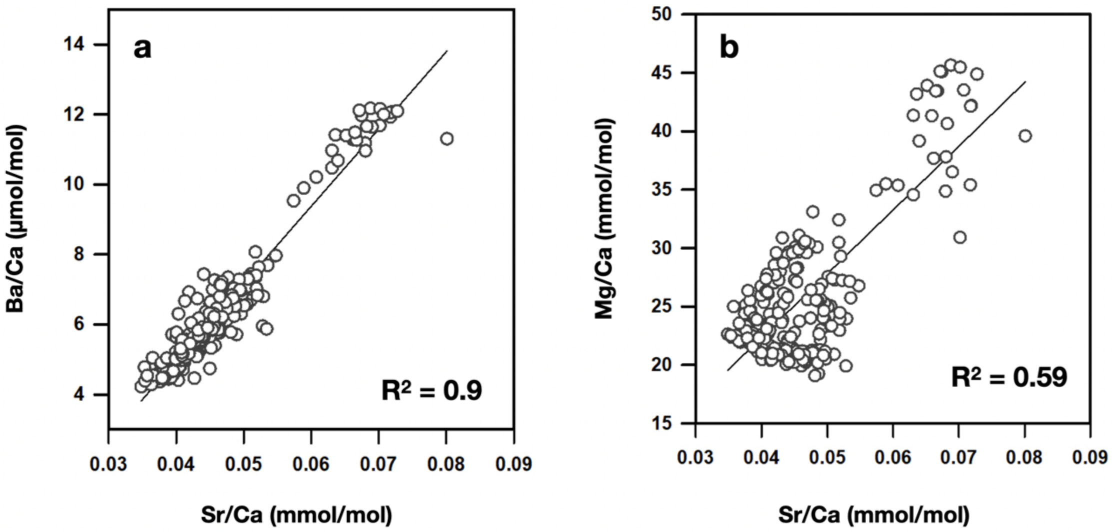

Supplementary Figure 5. Correlation analysis. (a) Correlation between Bàsura Ba/Ca and Sr/Ca ($r^2 = 0.90$, $n = 230$, $p < 0.01$). **(b)** Correlation between Bàsura Sr/Ca and Mg/Ca ($r^2 = 0.59$, $n = 232$, $p < 0.01$).

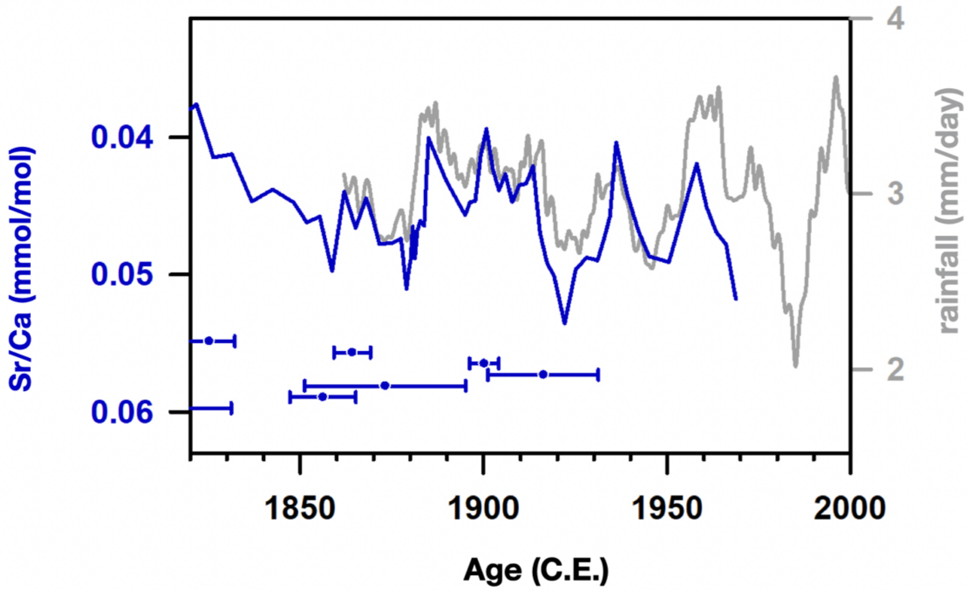

Supplementary Figure 6. Bàsura Sr/Ca and precipitation. The blue line shows the BA18-4 Sr/Ca ratio. The grey line shows the 10-year running average of Genoa/Milan/Nice rainfall. The Sr/Ca series was tuned to the rainfall data within age uncertainties with a correlation coefficient of -0.63 (n = 36, p < 0.01). Error bars indicate ^{230}Th dates with 2-sigma dating uncertainties.

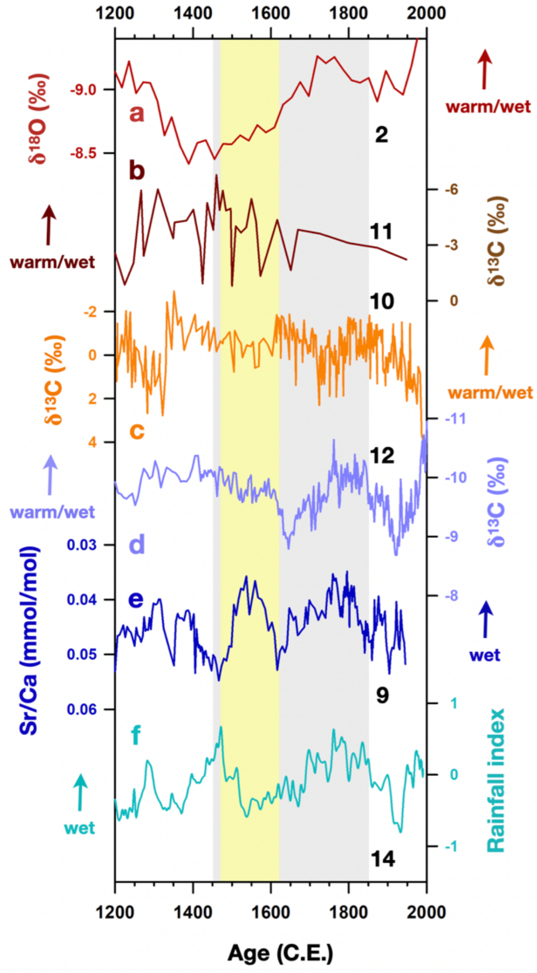

Supplementary Figure 7. Bàsura Sr/Ca and other records from Europe and North Africa. **(a)** Stalagmite $\delta^{18}O$ record from Sweden[40]. **(b)** Stalagmite $\delta^{13}C$ record from Portugal[43]. **(c)** Stalagmite $\delta^{13}C$ record from Spain[44]. **(d)** Stalagmite $\delta^{13}C$ record from Turkey[45]. **(e)** Bàsura Sr/Ca record from northern Italy (this study). **(f)** Stalagmite-based rainfall index reconstructed from Morocco[46] The upward arrows in **(a)**-**(f)** indicate a warm/wet climate.

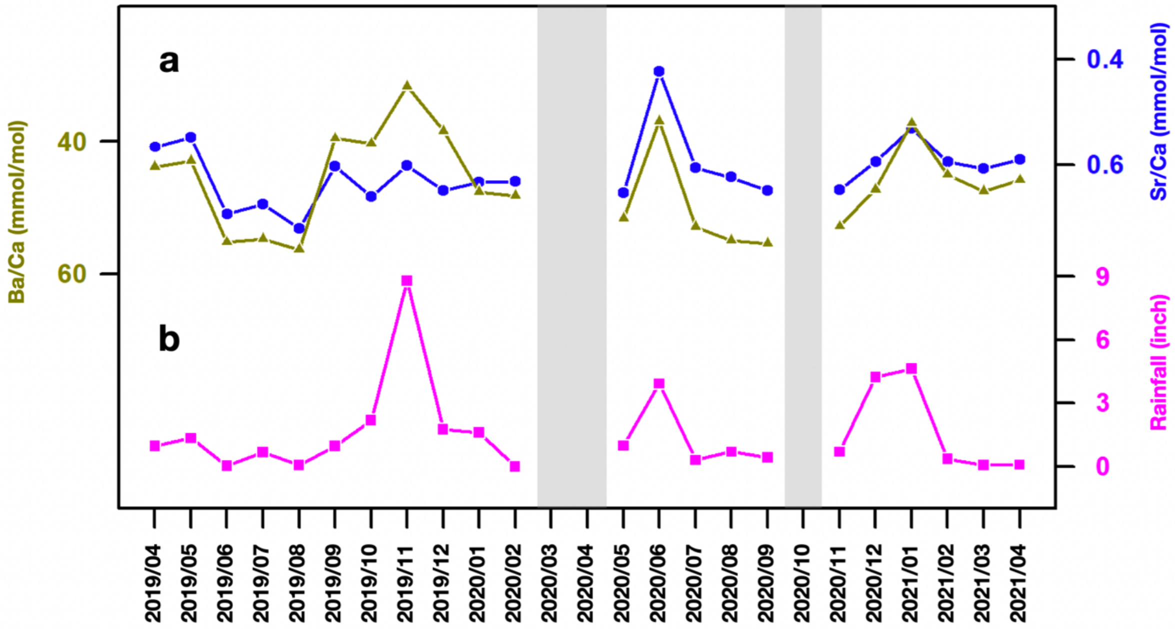

Supplementary Figure 8. In-situ monitoring of dripwater Sr/Ca and Mg/Ca at Bàsura cave. **(a)** Dripwater Sr/Ca (blue) and Ba/Ca (brown) at the chamber of BA18-4. **(b)** Rainfall data at Cape-Mele station (43.95 ˚N, 8.16 ˚E, 20 km south of Bàsura cave). The grey bars mark periods of missing data due to the COVID-19 outbreak.
