# Supplementary material for: Split westerlies over Europe in the early Little Ice Age
Source: Nat Commun. 2022 Aug 20;13:4898. doi: 10.1038/s41467-022-32654-w (PMC9392774; doi:10.1038/s41467-022-32654-w)
Supplement: Supplementary file 2 — Description of Additional Supplementary Files [file 41467_2022_32654_MOESM2_ESM.pdf]

Description of Additional Supplementary Files

File Name: Supplementary Data 1
Description: Mg/Ca, Sr/Ca and Ba/Ca of BA18-4
